# Fully electronic urine dipstick probe for combinatorial detection of inflammatory biomarkers

**DOI:** 10.4155/fsoa-2017-0142

**Published:** 2018-03-27

**Authors:** Vikramshankar Kamakoti, David Kinnamon, Kang Hyeok Choi, Badrinath Jagannath, Shalini Prasad

**Affiliations:** 1 Department of Bioengineering, University of Texas at Dallas, 800 W Campbell Rd, Richardson, TX 75080, USA

**Keywords:** EIS, inflammatory protein biomarkers, molybdenum affinity biosensor, portable electronic interface, urine dipstick probe

## Abstract

**Aim:**

An electrochemical urine dipstick probe biosensor has been demonstrated using molybdenum electrodes on nanoporous polyamide substrate for the quantitative detection of two inflammatory protein biomarkers, CRP and IL-6.

**Materials & methods:**

The electrode interface was characterized using ζ-potential and Fourier transform infrared spectroscopy. Detection of biomarkers was demonstrated by measuring impedance changes associated with the dose concentrations of the two biomarkers. A proof of feasibility of point-of-care implementation of the biosensor was demonstrated using a portable electronics platform.

**Results & conclusion:**

Limit of detection of 1 pg/ml was achieved for CRP and IL-6 in human urine and synthetic urine buffers. The developed portable hardware demonstrated close correlation with benchtop equipment results.

## Lay abstract

Early disease diagnosis helps in minimizing complications from various disease conditions. Quantitative readout of test results is useful in eliminating the error due to interpretation of output. In this paper, we have demonstrated the possibility of detection of two analytes in urine which holds promise to be developed as a home-based initial screening platform.

Urine, being one of the most easily accessible fluids of the human body, is an intriguing medium to investigate chronic noninvasive health conditions and infectious diseases due to the presence of numerous biomarkers in its composition [1,2]. Conventional laboratory procedures for urinalysis require long turnaround time and sophisticated instrumentation, which is undesirable [3]. However, in comparison, urinary dipstick-based biosensors have the advantages of rapid reporting, ease of manufacturing and low cost, making them attractive for use. However, commercially, these predominantly rely on qualitative colorimetric reporting mechanisms [4–6], while the literature does introduce some promising prospects in urinary biosensors, summarized in Table 1. The advancements reflected in these works have expanded the detection of identified traditional urinary biomarkers such as human chorionic gonadotropin (hCG), glucose and creatinine toward the detection of various other markers associated with health conditions such as liver and kidney function, sepsis, tuberculosis [7] and ZIKA virus [8–10]. The development of quantitative reporting of these versatile biomarkers is of significant interest for accurate interpretation of disease conditions in the human body.

**Table 1.** **Summary of urine-based biosensors for analyte detection.**

**Class**	**Biomarker**	**Detection method**	**Detection limit**	**Response time**	**Ref.**
Metabolite (noninfectious)	Glucose	Colorimetric	3e-7 mol l^-1^	20 min	[11]

	Glucose Lactate Uric acid	Electrochemical	0.35 mM 1.76 mM 0.52 mM	2 min	[3]

	HSA	Chemiluminescence	2.5 mg l^-1^	32 min	[12]

	hGH	Bimodal waveguide interferometry	10 pg ml^-1^	8 min	[13]

	Creatinine	Electrochemical	6.8 μg/dl	20 min	[14]

Pathogens (infectious)	*Schistosoma haematobium*	Electrochemical	0.53 ng/μl	60 min	[15]

	LAM	Optical	19 ng/ml	60 min	[16]

	*Escherichia coli*, *Neisseria gonorrhoeae*	Optical	10 CFU/ml	30 s	[17]

Protein (noninfectious)	NMP22	Electrochemical	3.3 pg/ml	N/A	[18]

	hCG	Electrochemical	2.4 pg/ml	30 min	[19]

	TGF-β1	Electrochemical	10 pg/ml	20 min	[20]

	Lactoferrin	Electrochemical	145 pg/ml	30 min	[21]

	IL-6, CRP	Electrochemical	1 pg/ml	5 min	This work

CFU: Colony-forming units; CRP: C-reactive protein; hGH: Human growth hormone; HSA: Human serum albumin; LAM: Lymphangioleiomyomatosis.

Quantitative detection of inflammatory biomarkers in urine assists in the early diagnosis of certain disease conditions. The detection of inflammatory biomarkers such as IL-6 and C-reactive protein (CRP) in urine hold significance in the preliminary detection of chronic diseases [22] as well as bacterial infections [23–25]. Urinary tract infections (UTIs), with inflammation being one of the primary symptoms, are among the most common bacterial infections with an annual healthcare expenditure of $3.5 billion in the USA [26,27]. The elevated levels of IL-6 in urine hold critical significance in the diagnosis of UTIs [28]. IL-6 is synthesized by T cells, endothelial cells, macrophages, B cells and fibroblasts after trauma and infection, resulting in inflammation. The local response of IL-6 occurs at the bladder mucosal surface as a response to bacterial infections [29]. The mean concentration of urinary IL-6 levels in healthy adults is approximately 3 pg/ml [30], while a concentration of 25 pg/ml is considered to be the threshold for requiring antibiotic treatment for UTIs [31]. Thus, detection of IL-6 in urine can serve as an initial screening tool aiding in the diagnosis of specific UTI infections.

The release of IL-6 triggers the liver to produce proteins such as CRP and fibrin. CRP is another key biomarker for diagnosis of inflammatory responses. It is recognized as a reliable method in differentiating bacterial and viral infections [32]. The mean concentration of urine CRP was established to be less than 150 pg/ml in healthy patients [33,34]. Both IL-6 and hs-CRP have been shown to have clinical utility as biomarkers for diagnosing UTI [35,36]. Hence there may be an advantage in combinatorial detection of IL-6 and CRP levels, as it would enable development of rapid point-of-care tests with a higher negative predictive value.

In this work, we present the development of a novel urinary diagnostic ‘dipstick probe’ biosensor, for combinatorial detection of inflammatory biomarkers: CRP and IL-6 through nonfaradaic electrochemical impedance spectroscopy (EIS). We leveraged the surface of a molybdenum (Mo) electrode on a flexible nanoporous polyamide substrate to develop an affinity assay for CRP and IL-6 detection using 1-ethyl-3-(3-dimethylaminopropyl) carbodiimide hydrochloride–N-hydroxysuccinimide (EDC–NHS) crosslinking chemistry. We have previously demonstrated the feasibility of Mo as a reliable electrode material for designing electrochemical biosensors [37]. The nanoscale network of intercalated pores of the polyamide membrane substrate allows for nanoconfinement of the target analyte enhancing the electrochemical response resulting from capacitive double-layer modulation [38]. The nanoporous structures also mitigate the charge screening effect caused by the nonspecific biomolecules [39]. The binding interactions of Mo with EDC–NHS crosslinker and antibodies were established using Fourier transform infrared spectroscopy (FTIR) and ζ-potential characterization techniques. In this work, we propose a direct measure of specific urinary analyte through a fully electronic transduction method. We have established the detection capability of our biosensor in physiologically normal and elevated levels of IL-6 and CRP in synthetic and human urine. At last, a proof-of-feasibility of a portable urinary dipstick probe electronic interface has been demonstrated. The aim of our work is to demonstrate the detection of inflammatory biomarkers in human urine for early disease detection.

## Materials & methods

### Materials & reagents

NHS, EDC, 2-(N-Morpholino)ethanesulfonic acid (MES; pH 3) and phosphate-buffered saline (PBS; 0.1 M, pH 7) were obtained from Sigma-Aldrich (MO, USA). Anti-CRP antibody, anti-IL-6 antibody and CRP antigen were procured from Abcam (MA, USA). IL-6 protein was procured from Thermo Fisher Scientific, Inc. (MA, USA). Pooled human urine samples were purchased from Lee Biosolutions, Inc. (MO, USA). Acrylic cellulose acetate sheets were obtained from Staples (TX, USA). Kapton backing support material was obtained from McMaster Carr (TX, USA). Polyamide membranes of 200 nm pore size were obtained from GE Healthcare (NJ, USA). All solvents and reagents were of analytical grade and used as received.

### Fabrication & surface characterization of Mo electrode

The electrode pattern was deposited onto the base polyamide substrate using a shadow mask thin-film electron-beam deposition process. The shadow mask stencils were made from acrylic cellulose acetate polymer sheets. A thin base layer of gold was deposited below the Mo to improve electrode adhesion. A secondary acrylic adhesive glue was patterned to prevent fluid wicking on to contact pads of the electrodes. The deposition profile of the deposited Mo was evaluated using scanning electron microscopy (SEM), energy dispersive x-ray spectroscopy (EDAX) and atomic force microscopy (AFM).

### FTIR characterization of surface chemistry

Attenuated total reflectance Fourier transform infrared (ATR-FTIR) spectrograms were performed to establish the binding of the EDC–NHS crosslinker to the Mo surface, and subsequent binding of IL-6 and CRP antibody to the EDC–NHS crosslinker. The tool was equipped with a deuterated triglycine sulfate detector and KBr window. A Harrick VariGATR sampling stage with a 65° germanium ATR crystal was used in this study. Measurements were taken as an average of 512 scans in the range of 4000–600 cm^-1^ with 4 cm^-1^ resolution. ATR-FTIR slides were prepared on cleaned glass slides with analogous metal deposition to the electrode fabrication process. The slides were incubated with 100 μl of the EDC–NHS mixture for 1 h. A 3 × PBS wash was performed to remove any unbound crosslinker. After washing and completely drying with nitrogen, the Mo EDC–NHS measurement was taken. After EDC–NHS functionalization, 100 μl of 10 μg/ml target antibody was incubated on the slide surface for an additional 30 min (one slide for IL-6 antibody and one slide for CRP). A 3× PBS wash was performed to remove unbound antibodies and the slides were dried with nitrogen and silica desiccant beads before the measurements were taken. All measurements represent individual slide preparations, with none being used more than once.

### Surface charge characterization with ζ-potential

ζ-potential measurements were performed to validate the construction of the immunoassay onto the Mo surface using Malvern Instrument's Zetasizer Nano ZS (MA, USA). Electrophoretic mobility technique was used for ζ-potential measurements. First, ζ-potential of Mo (VI) oxide nanoparticles (Sigma-Aldrich) was measured followed by EDC–NHS bound to the Mo nanoparticles. Further, ζ-potential of anti-IL-6 and anti-CRP antibodies bound to Mo EDC–NHS complex was measured. 1 ml of sample volume was used for each measurement. ζ-potential for IL-6 and CRP dose concentrations was performed in triplicate to validate the binding of immunoassay in synthetic urine at pH 6.45 between 10 pg/ml and 100 ng/ml using the assay developed on Mo surface. Smoluchowski approximation was used to compute the ζ-potential as given below:(1)
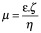



μ: Electrophoretic mobility; ε: Dielectric constant; ζ: ζ-potential; η: Viscosity of solution.

### Experimental protocol for biosensing experiments

A solution of 100 mM NHS and 400 mM EDC was dissolved in MES (pH 3) buffer just prior to incubation on the sensor. The resulting EDC–NHS complex was then incubated on the sensor surface for 1 h. Post-EDC–NHS functionalization, 8 μl of PBS was added to the sensing region to remove any unbound crosslinker. 8 μl of 10 μg/ml of monoclonal antibody (IL-6 or CRP) in 0.1 M PBS was incubated for 15 min. To emulate a true testing environment, test samples were prepared in spectrometer cuvettes containing 300 μl of sample, which is sufficient to fully cover the sensor when they are dipped in the cuvette. After approximately 15 s of being continuously dipped in the sample, the sensor was removed from the cuvette and allowed to incubate for 5 min. Studies were conducted in both synthetic urine and pooled human urine. The stability of Mo electrode to function efficiently in varying pH of urine was evaluated by performing electrochemical characterization studies in synthetic urine formulated with at pH 5.5, 6.5 and 7.5. A zero-dose measurement in the absence of target molecule was performed before introducing any samples. All dose concentrations of one target protein (either IL-6 or CRP) were tested on a sensor. IL-6 was tested in the range of 1 pg/ml–10 ng/ml, while CRP was tested in the range of 1 pg/ml–100 ng/ml mapping to their physiologically relevant ranges. EIS measurements were performed at each assay step on Gamry Reference 3000 potentiostat (PA, USA) and developed portable electronic reader in parallel. An AC voltage of 10 mV_RMS_ was applied with a frequency sweep of 1 Hz–1 MHz.

### Portable electronic dipstick interface

The developed electronic reader represented in Figure 5C and D demonstrated the translatability of the biosensor to a portable form factor. The device was constructed in its entirety from off-shelf components, and was programed using open-source code libraries on Arduino Uno R3 microcontroller. The sinusoidal excitation waveform was generated using an MCP4725 digital-to-analog converter. The current response at the working electrode was captured using a current-to-voltage amplification circuit (INA 121) and read back into the microcontroller using an ADS1015 analog-to-digital converter. The sensor consumes no more than 5 μA of current in a given measurement, and only takes 8–10 s to complete a measurement, making it an ideal transduction method for ultralow power biosensing.

## Results

### Surface characterization of Mo biosensor

A two-electrode electrochemical sensor was developed on porous polyamide (PA) substrate. The two-electrode system consists of a reference electrode which is curved around a circular working electrode at the center of the sensing region. The total area of the electrode was 0.7 cm^2^_._ The two electrodes were interfaced with test equipment using thin traces so that the sensing region of the electrode geometry dominates the impedance response. A COMSOL model of this electrode design included in Supplementary Figure 1, shows maximum distribution of current density and electric displacement field near the working electrode which is favorable for evaluating the current response from electrochemical characterization experiments [40].

**Figure 1.**
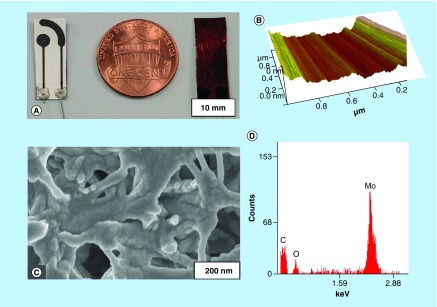
**Surface characterization of molybdenum biosensor.** **(A)** Two-electrode Mo biosensor with polyimide backing; **(B)** Atomic force microscopy image indicating uniform deposition profile of molybdenum; **(C)** scanning electron microscopy image indicating conformal deposition of molybdenum on polyamide substrate; **(D)** Energy dispersive x-ray spectroscopy characterization of scanning electron microscopy region in **(C)**. Mo: Molybdenum.

Further, surface characterization of the Mo-deposited electrodes was performed using SEM and AFM techniques to establish the uniformity of the metal deposition. Figure 1C shows the SEM micrograph of the Mo-deposited PA substrate, while Figure 1D shows the EDAX characterization of the represented micrograph. The results depict a strong presence of Mo as evidenced by peaks at 2.29 keV. The resistivity of the deposited Mo electrode measured with four-point probe source meter was 332 × 10^-3^ Ω m. To establish the uniformity of the deposition, AFM characterization (Figure 1B) was used. A low root mean square (R_q_) roughness of 2.36 nm was measured, with R_q_ value being an indicator of surface smoothness. With an Rq value of blank glass substrate being measured as 1.08 nm, change in the surface roughness after Mo deposition can be considered negligible. The baseline electrochemical characterization of Mo electrode was performed through open-circuit potential experiments in synthetic urine buffer. Mo electrode demonstrated a stable open-circuit potential for 10 min (data not shown) of measurement indicating the stability of the electrode in the absence of any applied input voltage.

### ATR-FTIR surface characterization analysis

**Figure 2.**
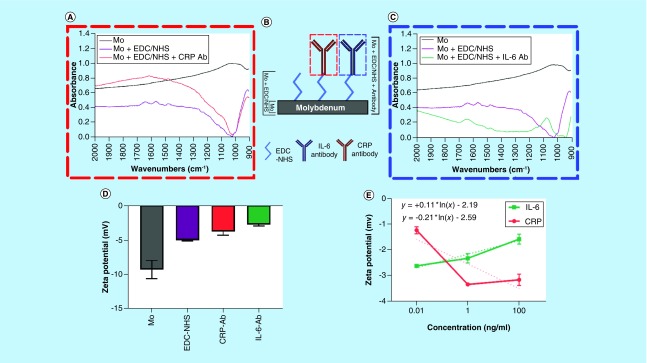
**Surface characterization of functionalized molybdenum sample.** **(A)** Fourier transform infrared spectroscopy (FTIR) spectra for functionalization of CRP; **(B)** Schematic representation of presented FTIR spectra; **(C)** FTIR spectra for functionalization of IL-6; **(D)** ζ-potential measurements for CRP and IL-6 antibodies; **(E)** ζ-potential characterization of CRP and IL-6 antigen concentrations. Ab: Antibody; CRP: C-reactive protein; EDC: 1-ethyl-3-(3-dimethylaminopropyl) carbodiimide hydrochloride; Mo: Molybdenum; NHS: N-hydroxysuccinimide.

ATR-FTIR was used to evaluate the nature of the chemical bonds present at the surface of the Mo electrode to validate the binding of both the EDC–NHS complex to the Mo surface as well as the subsequent binding of antibodies. Figure 2A shows a visualization of the three measurements taken to characterize the functionalization steps for each antibody. Figure 2B shows the resulting spectra. Table 2 outlines the associated wave number location for the relevant peaks in the figure. First, a measurement on blank Mo was done to characterize the presence of the Mo-native oxide, and to have a point of comparison for subsequent binding to the oxide surface. Next, the spectra postfunctionalization with the EDC–NHS crosslinker complex was taken, followed by measurements after the subsequent binding of IL-6 or CRP antibody. The spectra of the postcrosslinker and postantibody presented in Figure 2A and C are the result of subtraction from the Mo baseline to better visualize the peaks. The bare Mo sample shows an absence of discernible peaks with the exception of a broad peak manifesting from 900 to 1100 cm^-1^. This broad peak is characteristic of the Mo oxide layer that is leveraged for EDC–NHS binding. The FTIR spectra of the crosslinker and the antibody (Ab)-functionalized surfaces demonstrated signature peaks corresponding to the organic functional groups present in the respective molecules.

**Table 2.** **Fourier transform infrared spectroscopy characterization study of molybdenum and signature peak analysis.**

**Assignments**	**Peak positions (cm^-1^)**			

	**Mo**	**Mo + EDC–NHS**	**Mo + EDC–NHS + α-IL-6**	**Mo + EDC–NHS + α-CRP**
Moly oxide	900 – 1100	N/A	N/A	N/A

v (C=O)	N/A	1650	1647	1656

v_as_ (N–O)	N/A	1566	N/A	N/A

v_as_ (C–N, C–C)	N/A	N/A	1082	1047

CRP: C-reactive protein; EDC: 1-ethyl-3-(3-dimethylaminopropyl) carbodiimide hydrochloride; Mo: Molybdenum; NHS: N-hydroxysuccinimide.

### Surface charge characterization using ζ-potential

ζ-potential measurements can be used to quantify the modulation of surface charge due to the conjugation of charged species. Figure 2D shows that the ζ-potential of Mo nanoparticles in deionized water is -9.28 mV. The ζ-potential decreases to -5.01 mV due to the binding of EDC–NHS to Mo nanoparticles resulting in a change in surface charge. The surface charge modulation due to the binding of antibody–antigen results in the modulation of electrical double layer (EDL) [41].

For the CRP affinity assay, the ζ-potential after antibody conjugation is -3.73 mV as seen in Figure 2D. Figure 2E shows that the ζ-potential for three dose concentrations of CRP increases in magnitude from -1.24 to -3.35 mV for dose concentration of 10 pg/ml and 1 ng/ml, respectively. The slight drop in ζ-potential to -3.17 mV at 100 ng/ml of CRP may be attributed to the saturation of CRP. For the IL-6 affinity assay, the ζ-potential after antibody conjugation is -2.76 mV as seen in Figure 2D. Figure 2E shows that the ζ-potential for three dose concentrations of IL-6 decreases in magnitude from -2.6 to -1.5 mV with respect to increasing dose. This opposite behavior of CRP and IL-6 due to the varied modulation of EDL with varying dose concentrations is also seen in EIS measurements (discussed in ‘EIS characterization of CRP & IL-6’).

### EIS characterization of CRP & IL-6

EIS measurements were performed to electrochemically characterize the biosensing performance of the developed urine dipstick biosensor developed to detect CRP and IL-6 in urine.

#### EIS characterization of CRP & IL-6 in synthetic urine

When testing physiological buffers such as urine, it is important to first characterize the impact performance of the sensor under various physiological conditions such as pH variation. Urine is of variable pH across patients and hence, any affinity biosensor that is sampling for biomarkers in urine must be robust for biosensing over physiological range of urine. EIS biosensing experiments were performed on the developed Mo electrodes for CRP and IL-6 spiked in synthetic urine formulated according to the procedure described by Xu *et al*. [42] for pH 5.5, 6.45 and 7.5.

**Figure 3.**
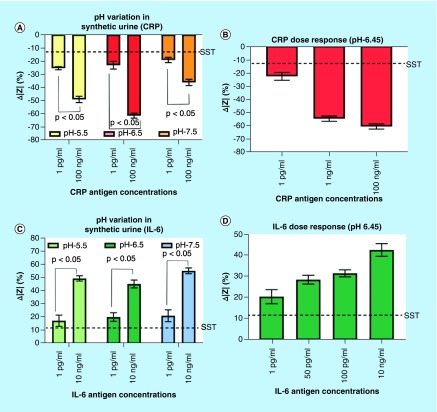
**Electrochemical impedance spectroscopy characterization of CRP and IL-6 in synthetic urine.** **(A)** Change in impedance analysis for low and high CRP antigen concentrations in three pH ranges of synthetic urine. Dotted lines indicate SST; **(B)** Impedance analysis for pH 6.45 synthetic urine; **(C)** Change in impedance analysis for low and high IL-6 antigen concentrations in three pH buffer ranges of synthetic urine. The dotted lines indicate SST; **(D)** Change in impedance analysis for four IL-6 antigen concentrations in pH 6.45 buffer of synthetic urine. CRP: C-reactive protein; SST: Specific signal threshold.

Figure 3A represents the percentage change in impedance at 1 Hz frequency for low (1 pg/ml) and high (100 ng/ml) CRP concentrations spiked in synthetic urine of pH 5.5, 6.5 and 7.5. A decreasing trend in impedance change was observed for increasing dose concentrations of CRP, approximately -20% and approximately -60% for 1 pg/ml and 100 ng/ml, respectively, across the pH ranges tested. The percentage change in impedance for low and the high CRP dose concentrations demonstrated statistical significance of p < 0.05 at 95% confidence interval using *t*-test for all three pH buffers of the synthetic urine, thereby indicating the sensitivity of Mo electrode to detect CRP in varying ionic conditions of urine. Figure 3B shows the calibration dose response for CRP using synthetic urine at pH buffer 6.45, similar to the pH of pooled human samples as described in section 4.4.1. The change in impedance ranged from approximately -22% for 1 pg/ml CRP concentration to approximately -61% for 100 ng/ml CRP concentration, and was governed by the equation: Δ|Z| = -3.451*ln[CRP in ng/ml] – 48.82 demonstrating an R^2^ value of 0.932. A specific signal threshold (SST) of 13% was computed using the following formula.(2)
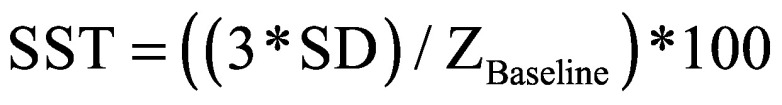



A limit of detection (LOD) of 1 pg/ml was obtained which is the smallest concentration above SST. Figure 3C shows the percent change in impedance at low (1 pg/ml) and high (10 ng/ml) concentrations of IL-6 across varying pH. The percentage change in impedance for 1 pg/ml of IL-6 antigen was calculated to be approximately 20% and approximately 50% for 10 ng/ml at all three pH ranges. This presents an opposing trend to the impedance response observed in Figure 3A and B for CRP, which is in agreement with the ζ-potential data presented in Figure 2. Figure 3D shows the calibration dosing response for 1 pg/ml to 10 ng/ml IL-6 in pH 6.45 synthetic urine, and was governed by the equation: Δ|Z| = 2.405*ln[IL-6 in ng/ml] + 36.20 demonstrating an R^2^ value of 0.995. All dose responses were above the measured SST of 11% with LOD of 1 pg/ml. These results help to establish that the sensor is robust to varying pH and is suitable for testing in physiological buffer. The stability of the antibody functionalization on sensor surface was established through multiple buffer wash steps post Ab functionalization. The impedance values of the subsequent buffer wash steps were statistically insignificant (p > 0.05). The plot has been shown in Supplementary Figure 2A. The specificity of the assay was demonstrated with the addition of creatinine on an IL-6 antibody-functionalized sensor and the results are included in the supplementary information (Supplementary Figure 2B). The absence of correlation between the changes in the impedance with increasing creatinine concentrations spanning its physiologically expressed range established the specificity of the developed assay.

#### EIS characterization of CRP & IL-6 in human urine

After establishing the sensor's performance in synthetic urine, the sensor was tested using spiked samples of IL-6 and CRP in pooled human urine to evaluate the electrochemical response of the biosensor, and to demonstrate translatability of the technology to complex human samples.

**Figure 4.**
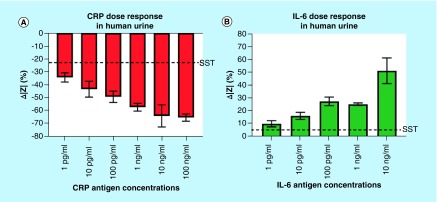
**Electrochemical impedance spectroscopy characterization of CRP and IL-6 in human urine.** **(A)** Change in impedance analysis for CRP antigen concentrations; **(B)** Change in impedance analysis for IL-6 antigen concentrations. Dotted lines indicate SST. CRP: C-reactive protein; SST: Specific signal threshold.

Figure 4A indicates the percentage change in impedance for CRP antigen concentrations from 1 pg/ml to 100 ng/ml. An SST of 21% was calculated for the CRP detection assay. The LOD was therefore determined to be at 1 pg/ml, as it is still detected over the established SST. The LOD is within the normal levels of CRP observed in urine. The change in impedance ranges from -34 to -65% increasing as a function of C_dl_ modulation with increasing CRP concentrations. Figure 4B shows the change in impedance for the IL-6 antigen concentrations from 1 pg/ml to 10 ng/ml. The LOD for the IL-6 detection was also determined to be 1 pg/ml, which is in the normal physiological range of IL-6 in urine. The change in impedance ranged from 10 to 51% and is above the 5% SST established for this study. The governing equation for the linear dynamic range from 1 pg/ml to 10 ng/ml is: Δ|Z| = 3.970*(IL-6 in ng/ml) + 34.95 demonstrating an R^2^ value of 0.844.

### Comparison of impedance response with portable electronic hardware prototype

To demonstrate the translatability of the developed dipstick probe assay into a true portable point-of-care device, a portable reader was developed. The device was designed to perform single frequency EIS measurements at the established 1 Hz analysis point used for detection of the target proteins on the benchtop instrument. The device was designed to capture the changes in impedance response of the sensor from the postfunctionalization baseline to the selected dose regimes of either CRP or IL-6 to the same level of sensitivity as can be attained by the benchtop instrument.

**Figure 5.**
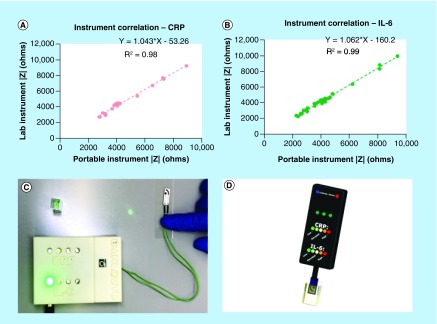
**Performance of portable electronic device.** **(A)** Impedance correlation between lab instrument and portable instrument for CRP; **(B)** Impedance correlation between lab instrument and portable instrument for IL-6; **(C)** Visual demonstration of test results using electronic interface; **(D)** 3D model representation of portable urine dipstick probe interface. CRP: C-reactive protein.

To quantifiably establish comparability of the device's measurements to the benchtop instrument, the raw impedance values of the device were plotted against the corresponding impedance values of the benchtop instrument for the detection of both molecules. This correlation is plotted for CRP in Figure 5A, showing a linear response with an R^2^ value of 0.98 with a slope near 1.0. The governing equation can be defined as y = 1.043x – 53.26. Figure 5B shows the same correlation when detecting IL-6, showing a linear response with an R^2^ value of 0.99 with a slope near 1.0. The governing equation can be defined as y = 1.062x – 160.20. These results show that the device is sensitive to the range of impedances produced by the sensor showing comparable performance to the benchtop instrument. Figure 5C shows the physical device and demonstrates an example of reporting elevated levels of IL-6 (1–100 pg/ml), while Figure 5D shows the envisioned commercial implementation of this device.

## Discussion

The surface characterization experimental results established that there is a uniform and smooth deposition of Mo on the nanoporous polyamide substrate. The FTIR analysis of the blank Mo substrate indicated the presence of a passive oxide layer which is leveraged for binding of EDC–NHS crosslinker molecules to the electrode surface. After EDC–NHS binding, the peak seen at 1650 cm^-1^ is indicative of the v (C=O) carbonyl stretching of the EDC–NHS. The carbonyl stretching is expected postantibody conjugation and is observed in both antibody measurements (1647 and 1656 cm^-1^ for IL-6 and CRP antibody, respectively). The peak at 1566 cm^-1^ observed in the EDC–NHS measurement is from v_as_(N–O) nitro stretching of the crosslinker, which disappears in the antibody measurements after the aminolysis of the NHS after binding. Once the antibody binds, a new bond v_as_(N–C, C–C) can be seen at 1082 and 1047 cm^-1^ for IL-6 and CRP antibody, respectively. This evidence suggests that EDC–NHS has strongly bound to the Mo oxide layer, and is stable through the functionalization steps associated with the assay. The results also indicate that both the IL-6 and CRP antibodies are binding to the EDC–NHS crosslinker at the surface of the Mo as opposed to other mechanisms such as physical absorption.

The ζ-potential measurements indicate the surface charge changes and hence we leveraged this property toward characterizing the modulation of the EDL due to antibody–antigen binding of IL-6 and CRP on the Mo nanoparticle surface. ζ-potential is also a measure of repulsion behavior of identical charged species. As a larger molecule (105 kDa), the number of identically charged species is greater at higher dose concentrations of CRP, thus, the ζ-potential increases with increasing dose concentrations. Since, IL-6 is relatively small based on molecular weight (26 kDa), the number of IL-6 molecules docking on to individual Mo surface would be higher at higher concentration. This results in a lower surface charge, thus, reducing the ζ-potential at higher concentrations. The opposing trends in ζ-potential with respect to concentrations of IL-6 and CRP are due to the difference in the binding mechanism of both the biomolecules on the Mo surface, thus, modulating the EDL differently. EDL consists of two layers, in other words, stern layer and diffuse layer. There exists a ‘slipping plane’ within the diffuse layer where the potential drops linearly from the stern plane. Beyond the slipping plane, the potential drops exponentially. The potential at the slipping plane is the ζ-potential. Higher molecular weight of the macromolecular species results in the slipping plane at a larger distance from the stern plane [43]. The higher ζ-potential of CRP as compared with IL-6 can thus be attributed to the higher molecular weight of CRP. As ζ-potential is a measure of EDL, it can be used to correlate with techniques that modulate the EDL with small voltage perturbations [41].

The changes in impedance due to biomolecular binding of CRP and IL-6 to the corresponding capture probes at the electrode/solution interface were analyzed through EIS. The primary objective was to evaluate the electrochemical response of the developed biosensor across varying pH of urine. Nonfaradaic EIS primarily captures the EDL dynamics at the electrode–solution interface due to small voltage perturbations. The effect of solution resistance is a surface phenomenon and is typically dominated at higher frequencies of the impedance spectrum [44]. The change in impedance within the EDL is mainly due to modulation of the C_dl_ resulting from the binding between specific antibody and antigen interaction. The impedance response with varying concentrations of the target analyte were analyzed at 1 Hz frequency due to the dominance of C_dl_ at low frequencies [44,45]. Also, maximum change in impedance response for multiple antigen concentrations was observed at 1 Hz frequency and hence it was chosen as the frequency of analysis of impedance spectra. The negative change in impedance for the CRP dose concentrations indicates that the impedance of dose concentration increases from the baseline impedance. This phenomenon can be attributed to the charge state of CRP in the urine buffer. The dipstick probe biosensor exhibits a wide dynamic range that spans over normal and elevated levels for both CRP and IL-6 in human urine samples, thereby establishing its usefulness to be used as an electronic dipstick probe biosensor.

The goal of the portable electronic reader was to establish that the device was sensitive to the range of impedances exhibited by the sensor, and that the device reports those impedance values with the same accuracy as the benchtop instrument. The main source of error in the correlation of the two tools came from fluctuations in solution resistance as function of time when reconfiguring the sensor from one tool to another, which is not a factor of the device itself. Overall, the device demonstrates translatability of the urinary dipstick for inflammatory biomarkers toward a true point-of-care diagnostic form factor.

## Conclusion

An affinity-based electrochemical biosensor for the detection of two critical inflammatory biomarkers, namely CRP and IL-6, was developed using Mo electrode on a nanoporous flexible substrate. The surface characterization performed through SEM analysis demonstrated a uniform deposition of Mo on the polyamide substrate. The presence of peaks, in FTIR spectra, correlated with the EDC–NHS crosslinker molecules, thereby establishing the surface functionalization of Mo electrode with crosslinker monolayer. Binding of CRP and IL-6 antibody with EDC–NHS-functionalized Mo surface was confirmed using FTIR characterization. The change in the surface charge, calculated through ζ-potential measurements, correlated to the antigen concentrations, thereby validating antigen binding to crosslinker–antibody conjugate. The affinity-based antigen–antibody binding was characterized through nonfaradaic EIS. The dipstick probe biosensor demonstrated ability to detect CRP and IL-6 antigen concentration as low as 1 pg/ml in both pooled human urine and synthetic urine samples. The detection concentration range correlated with threshold cut-off concentrations of diseases associated with these biomarkers. The sensor's performance was preserved in multiple pH range of synthetic urine. This work is the first demonstration of combinatorial detection of CRP and IL-6 in urine using an electrochemical dipstick probe with Mo electrode. The portable research interface established a close correlation with benchtop measurements thereby supporting its development as a semiquantitative point of care biosensor for near-patient testing for detecting inflammatory biomarkers.

## Future perspective

The current Mo electrode nanoporous dipstick probe biosensor has the capability to be developed as a quantitative error-free reporting platform for biomarker analysis in human urine. The biosensor holds potential for detecting multiple biomarkers from a single sample. The portable electronic hardware is envisioned as a point-of-care diagnostics tool to be used in clinics and in a home environment. The stability of the electrode enables dynamic monitoring of urinary biomarkers over multiple time intervals thereby benefiting patients with chronic health conditions.

**Summary points**

- Label-free electrochemical urine dipstick probe with molybdenum electrode has been developed for combinatorial detection of IL-6 and C-reactive protein in urine.
- The performance of the electrode to detect the target biomarkers has been evaluated in synthetic urine with multiple pH buffers and in human urine.
- The feasibility to perform point-of-care detection in urine has been demonstrated with a portable electronic research hardware.
- Distinguishability between normal and elevated levels of C-reactive protein and IL-6 in urine has been demonstrated.
