# Supplementary material for: Fully electronic urine dipstick probe for combinatorial detection of inflammatory biomarkers
Source: Future Sci OA. 2018 Mar 27;4(5):FSO301. doi: 10.4155/fsoa-2017-0142 (PMC5961415; doi:10.4155/fsoa-2017-0142)
Supplement: Supplementary file 1 [file fsoa-04-301-s1.docx]

**Supplementary information:**

**COMSOL simulation of current density and electric displacement field distribution:**

The electric displacement field and current density distribution of the applied voltage was simulated using the in-built Electrostatics and Electric current physics equations. The current density plot indicated a maximum current density distribution near the working electrode. The electric displacement field also demonstrated a maximum distribution near the working electrode thereby indicating that the response from the EIS is from the working electrode.


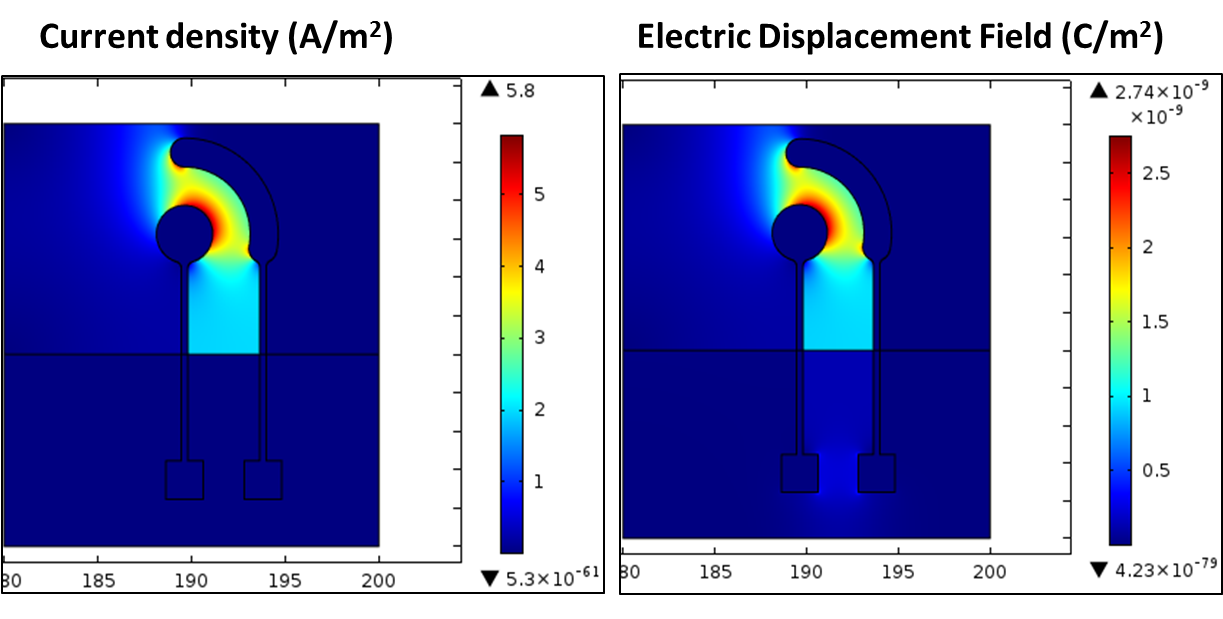


Figure S1 COMSOL simulation of current density and electric field displacement field distribution between working and reference electrode geometries.

**Assay stability study with buffer wash steps:**

The stability of the assay was evaluated by performing multiple buffer washes post IL-6 Antibody functionalization step. The impedance of buffer wash steps were studied at 1 Hz. The changes in impedance of the sensor for the buffer wash steps were statistically insignificant (p>0.05) even after 5 washes thereby indicating the stability of the antibody functionalized on the sensor surface (Figure S1 A).


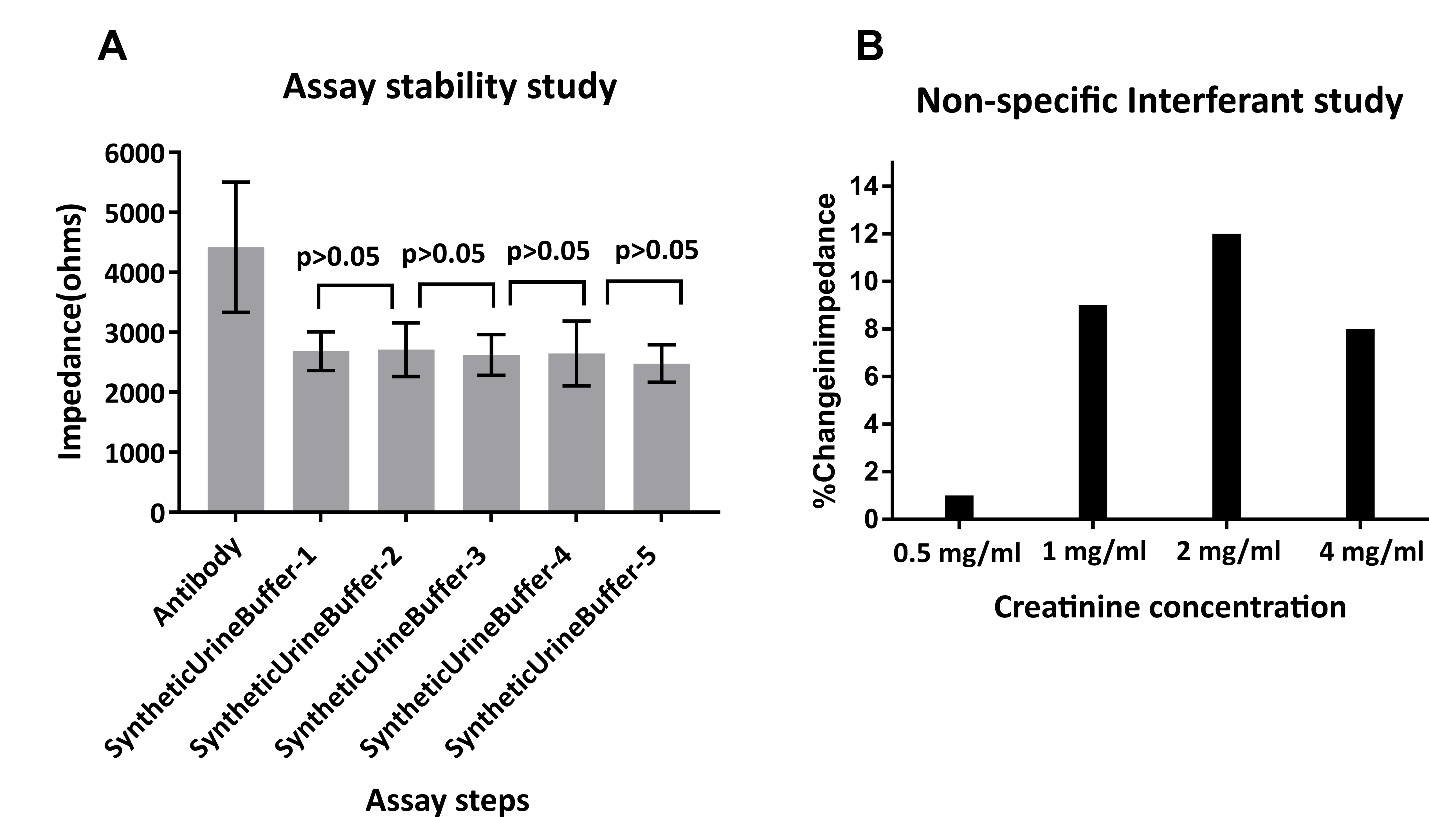


Figure S2 (A) Assay stability study with multiple blank buffer washes post Ab functionalization (B) Non-specific interferant study with creatinine on IL-6 Antibody functionalized sensor.

**Non-specific interaction study:**

The specificity of the assay was evaluated by performing non-specific interaction study with Creatinine which is a major interfering agent in urine. The valid concentrations of Creatinine as recommended by WHO is from 30 mg/dL to 300 mg/dL[1]. The urinary concentrations ranging from 0.5 mg/ml to 4 mg/ml were tested on IL-6 functionalized sensor. The sensor demonstrated a negligible change in impedance with respect to baseline impedance value. The absence of correlation between Creatinine concentrations with change in impedance values establish the absence of binding between non-specific creatinine molecules with IL-6 antibody functionalized sensor surface. Thus, the response of the sensor to non-specific molecules in urine was established.

**References:**

1. Barr DB, Wilder LC, Caudill SP, Gonzalez AJ, Needham LL, Pirkle JL. Urinary creatinine concentrations in the US population: implications for urinary biologic monitoring measurements*.* *Environmental health perspectives* 113(2), 192 (2005).
